# Failure to treat- how a broken healthcare system puts patients and practitioners at risk^⋆^

**DOI:** 10.1016/j.ipej.2025.12.014

**Published:** 2025-12-08

**Authors:** Sanjeev Saksena

**Affiliations:** a Rutgers’- Robert Wood Johnson Medical School, New Brunswick, NJ, USA; b Electrophysiology Research Foundation, Warren, NJ, USA

**Fig. 1:**
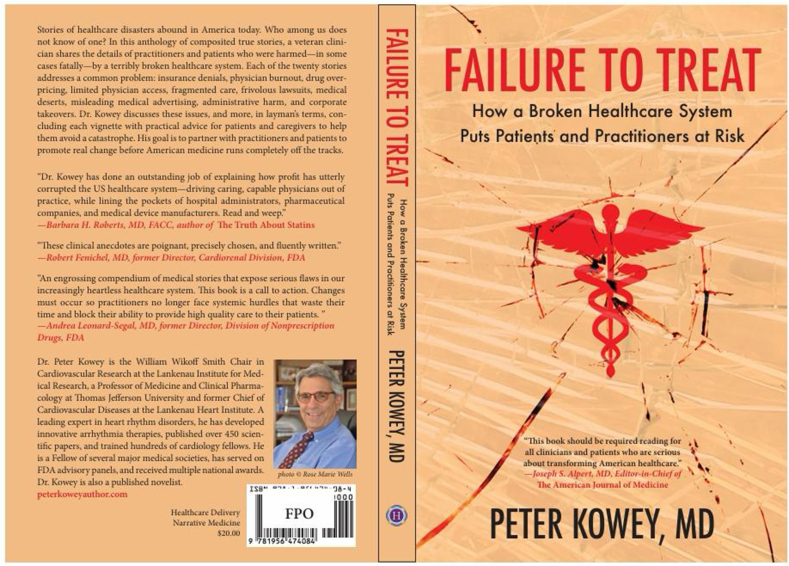
Book title - Failure to Treat, Author - Dr Peter Kowey.

In this book (Fig. 1), Dr. Peter Kowey takes a long, measured and critical view of the United States Health Care system in its formulation at the end of the first quarter of the 21st century. He does this through the lens of delving into his patients' personal stories, as they encounter and navigate the health care system. This long look spans a half century of involvement in the system, in which Dr. Kowey has risen to the heights achievable for a medical practitioner, scholar, researcher and clinical investigator in cardiology and electrophysiology. Mentored by some of the finest minds in cardiology of the 20th century, he writes this book at the behest of one of them, Professor Bernard Lown, Nobel Laureate, Harvard Professor, Pioneer and Cardiologist *Extraordinair*e. In Peter's own foreword to this book, he sets the scene for the reader when he writes:

*“Dr. Lown’s greatest achievements were at his patient’s bedside where he made it his habit to heal hearts and to mend souls. Much of this book emanates from conversations I had with Dr. Lown in his final months. By exposing every part of the scandal that medicine has become, I hope that healthcare providers can begin the process of resuscitating our beloved profession, and our patients can more successfully navigate their way to better health”*

In 20 patient vignettes, based both in hospital and the clinic, he exposes daily medical and structural dilemmas in the US medical system. Each is written in lay prose for the public, initially outlining the medical condition of the patient with simple and clear explanations. This direct outreach to the final recipient of health care, the public, is a strong suit of this book. Following a trend originating in the nineteen eighties in Cardiology, with books such as “*Open Heart Surgery: A guidebook for patients and families* [1], Dr. Kowey has chosen to speak directly to the underwriter and consumer of health care, the US public. For the health care practitioner, a central link in this, this book is indispensable as they look to the future. In emerging nations, yet to encounter all the pitfalls outlined, it is cautionary tale.

Kowey takes the reader behind the scenes into the medical system pathways that handle the patient's medical condition. He dissects the administrative and economic compulsions arising from intense influence of health care business ownership, insurance company control of reimbursement, private finance entrepreneurship and return on investment in pharmaceutical and medical device industries, and innumerable middle men and brokers that succeed in driving up costs to the health care system. In doing so, a well-meaning national social security net, designed to provide health care to the elderly and the indigent, becomes entangled in this mesh. Despite repeatedly reinventing itself to changes and advances in medicine and physician practice, the Government health system is unwittingly compensating, collaborating and tolerating this behavior and inefficient use of its resources.

Dr. Kowey paints with a wide ranging brush, and few aspects of the system escape his review. Currently lauded great advances in health care delivery from electronic medical records, medical technology and direct to consumer advertising are all featured. The Achilles’ heels of each of these and many other aspects of the new health care milieu are many, often resulting in devastating patient outcomes. He steps into regulatory agency behavior and medical education missteps and finally, most critically, talks to physician burnout becoming the final fallout of these new practices in the medical environment. Kowey goes beyond defining the missteps and breakdowns in the current health care system. For the layman, he provides an explanation of individual cases that resulted in the observed consequences.

For readers of this Journal, the takeaway messages are many, but some standout. Firstly, electronic information systems are far from their overblown claims of improved communication and portability of knowledge. They produce gargantuan amounts of data, rarely perused, but do allow for increasing billing and charges to the reimbursement system. Now clinically inefficient, cumbersome and user unfriendly, they contribute to fragmentation of care, as direct human contact between health care providers and their patients evaporates in the electronic environment. Secondly, that health care funding and support determines Medicine's culture and work environment. Thirdly, privatizing reimbursement via insurance companies actually adds to costs, and reduces delivery of health care to the patient in actual dollars by the for-profit entity. Fourthly, despite its many contributions to improved medical care, unbridled technology promotes and profits from the business model of health care. In an entrepreneurial and uncontrolled private business model, the culture of Medicine is perverted by inappropriate incentives. The final outcome, connection and communication between physician, patient and the public is increasingly disrupted and patients have few advocates.

Arnold Relman, when delivering the Shattuck lecture in the 1980s, warned of the consequences of the medical-industrial complex [2]. A decade later, in the same oration observed that in that decade, 70–80 % of hospitals were non-profit at its beginning and over 85 % were for profit at its end [3]. Similarly, Dr. Kowey, in defining the missteps, challenges and failures in the current system, has opened the door to the ultimate solution(s). An informed educated public, in combination with knowledgeable health care practitioners, who see the larger and smaller pictures in a complex health care system, will have the tools and the ultimate charge to refashion and redirect the health care expenditures in their nation(s). This book takes an early step in this direction. For the electrophysiologist/cardiologist reader of this Journal, some time ago I proffered a Latin phrase “Festina Lente” (hasten slowly), as a guide to future actions [4]. It is a curiously apt aphorism for -our cutting edge, technology-driven discipline of medicine.

## Declaration of competing interest

The authors declare that they have no known competing financial interests or personal relationships that could have appeared to influence the work reported in this paper.
